# Effect of Instant Controlled Pressure Drop (DIC) Treatment on the Detection of Nut Allergens by Real Time PCR

**DOI:** 10.3390/foods9060729

**Published:** 2020-06-03

**Authors:** Africa Sanchiz, Carmen Cuadrado, Joseph Haddad, Rosario Linacero

**Affiliations:** 1 Food Technology Department, SGIT-INIA, Ctra. La Coruña Km. 7.5, 28040 Madrid, Spain; africa.sanchiz@inia.es (A.S.); cuadrado@inia.es (C.C.); 2 Laboratory Engineering Science for Environment (UMR 7356 CNRS), La Rochelle University, Avenue Michel Crepeau, 17042 La Rochelle, France; j.haddad@ul.edu.lb; 3 Food Science and Technology Department, Faculty of Agriculture, Lebanese University, Dekweneh, 11111 Beirut, Lebanon; 4 Genetics, Physiology and Microbiology Department, Biology Faculty, Complutense University, 28040 Madrid, Spain

**Keywords:** real-time PCR, tree nuts, allergen detection, processed foods, thermal processing, pressure processing, DIC processing

## Abstract

Tree nuts show nutritional properties and human health benefits. However, they contain allergenic proteins, which make them harmful to the sensitised population. The presence of tree nuts on food labelling is mandatory and, consequently, the development of suitable analytical methodologies to detect nuts in processed foods is advisable. Real-Time PCR allowed a specific and accurate amplification of allergen sequences. Some food processing methods could induce structural and/or conformational changes in proteins by altering their allergenic capacity, as well as produce the fragmentation and/or degradation of genomic DNA. In this work, we analysed by means of Real-Time PCR, the influence of pressure and thermal processing through Instant Controlled Pressure Drop (DIC) on the detectability of hazelnut, pistachio and cashew allergens. The detection of targets in hazelnut, pistachio and cashew (Cor a 9, Pis v 1 and Ana o 1, respectively) is affected by the treatment to different extents depending on the tree nut. Results are compared to those previously obtained by our group in the analysis of different treatments on the amplificability of the same targets. Reduction in amplificability is similar to that reported for some autoclave conditions. Our assays might allow for the detection of up to 1000 mg/kg of hazelnut, pistachio and cashew flours after being submitted to DIC treatment in food matrices.

## 1. Introduction

Food allergy is a worldwide health concern that affects around 2% of the population, reaching 8% in children [1]. The most common foods associated with food allergy are fish, peanuts, soybean, milk, eggs, crustaceans, wheat and tree nuts. Tree nuts are often responsible for severe and even lethal reactions on sensitized patients [2] and, according to the European regulation, they are one of the 14 allergenic ingredients whose presence must be indicated on the food label (Regulation (EU) 1169/2011). Most tree nut allergens are identified as seed storage proteins such as vicilins, legumins and albumins [3]. Hazelnut allergy represents an important health problem in industrialised countries because of its high prevalence and severity [4]. Several hazelnut allergens are characterised, such as Cor a 1 (Bet v 1 family), Cor a 2 (profilin), Cor a 8 (lipid transfer protein, LPT), Cor a 9 (11S globulin), Cor a 11 (vicilin-like protein), Cor a 12, Cor a 13 and Cor a 14 (2S albumins). Cashew nuts are responsible for serious allergic reactions in sensitive patients, frequently involved in anaphylactic responses [5]. Three major allergens have been described: Ana o 1 (7S vicilin), Ana o 2 (11S globulin) and Ana o 3 (2S albumin). Finally, five allergens are characterised in pistachio, including Pis v 1 (2S albumin), Pis v 2 and 5 (11S globulin), Pis v 3 (7S vicilin) and Pis v 4 (SOD) [6].

To date, there is no treatment for food allergies, so patients must avoid any contact and consumption of allergenic ingredients. Thus, available sensitive and specific detection methods of hidden allergens in foods are essential in order to protect sensitized individuals and comply with current regulations. Enzyme-Linked Immunosorbent Assay (ELISA) is the most-used methodology for direct detection of proteins from specific foods, as well as other immunoassays, such as lateral flow. Many studies have demonstrated that certain thermal treatments might be useful to diminish the allergenic potential of nuts, influencing allergen structure [7,8]. The solubility of allergenic proteins is often affected by thermal treatments [9] and, as a consequence, the performance of protein-based detection methods, as ELISA, might be highly compromised. Moreover, cross-reactivity is often referred to in this technique [10]. Alternatively, DNA-based methodologies have been developed in the last years for many tree nuts such as indirect detection approaches, especially Real-Time PCR, due to its sensitivity and specificity. In the last years, other trends in food allergen detection include biosensors, (especially immunosensors and genosensors), which are advantageous for the development of lab-on-chip systems, allowing the fast, sensitive and inexpensive analysis of samples [11]. DNA molecules are normally more stable to chemical and thermal processing and can be more efficiently extracted from raw and processed samples. For this reason, DNA-based assay is becoming a promising alternative to proteins for allergen detection in highly processed food samples [10]. To develop and validate the suitable performance of a Real-Time PCR assay for food allergen detection, the influence of thermal and non-thermal treatments on the target isolation and amplification should be analysed [12]. Particularly, for tree nuts, Real-Time PCR has resulted in a specific, reliable and sensitive method for the detection of many allergens [11].

Foods, including tree nuts, are frequently submitted to thermal and non-thermal processing in the food industry, to improve their organoleptic properties or ensure their safety [13]. Roasting, boiling, baking or frying are common thermal treatments applied in the food industry. Some of these thermal treatments, such as autoclave, are applied in combination to pressure, resulting in a reduction of immunoreactivity of several tree nuts [14,15,16]. Instant Controlled Pressure Drop (DIC) [17] is also a pressure-based thermal treatment that has been applied to foods trying to diminish their allergenic potential, especially in legumes [18,19]. 

Before this study, the development and validation of Real-Time PCR methods for pistachio, cashew and hazelnut were performed and published [20,21,22]. In the three works, allergen-coding genes were selected as targets (Pis v 1 for pistachio, Ana o 1 for cashew and Cor a 9 for hazelnut). Moreover, the detection of targets Pis v 1, Ana o 1 and Cor a 9 and the influence on their amplification have been analysed in different samples processed by boiling, autoclave, roasting or high-hydrostatic pressure (HHP). In this work, we have performed binary mixtures (control and treated flours in wheat flour) and the same DNA isolation method for all the samples. We tested the effect of a specific thermal and pressure treatment, instantaneous controlled pressure-drop (DIC), on the amplificability and performance of three validated Real-Time PCR systems, using the same published primers and probes.

## 2. Materials and Methods 

### 2.1. Plant Material

Three nut seeds have been used in this study: hazelnut, pistachio, and cashew. Pistachio (*Pistachia vera* Kerman) and hazelnut (*Corylus avellana*, Negreta) were obtained from the Germoplasm Bank of Institut de Recerca i Tecnologia Agroalimentaries (IRTA-Mas de Bover, Tarragona, Spain), whereas cashew nuts (*Anacardium occidentale*, type 320) were provided by Productos Manzanares S.L. (Cuenca, Spain). 

### 2.2. Instantaneous Controlled Depressurization Treatment (DIC)

Whole cashew, peanut, pistachio and hazelnut seeds were subjected to controlled Instantaneous Depressurization (DIC) treatment, performed at the La Rochelle University (LaSiE). DIC treatment was carried out following a factorial experimental design previously described [17]. In this experiment, the moistened whole nuts are placed in a processing chamber and exposed to steam pressure (7 bar) at high temperature (up to 170 °C), over a short time (120 s). An instant pressure drop towards a vacuum at about 50 mbar follows this high-temperature-short time stage. This abrupt pressure drop, at a rate ΔP/Δt higher than 5 bar s^−1^, simultaneously provokes an auto-vaporization of a part of the water in the product, and an instantaneous cooling of the products, which stops thermal degradation.

### 2.3. Binary Mixtures

Untreated and DIC treated nut seeds were ground with a kitchen robot (Thermomix 31-1, Vorwerk Elektrowerke, GmbH & Co. KG, Wüppertal, Germany) and defatted with n-hexane (34 mL/g) for 4 h, followed by air-drying. Flours were passed through a 1 mm mill and stored at 4 °C until use.

Several binary mixtures (10, 100, 1000, 10,000, 100,000 mg/kg) were obtained by mixing known amounts of treated defatted nut flour (from Kerman pistachio variety, type 320 cashew and Negreta hazelnut variety) in spelt wheat (*Triticum spelta* L.) flour, in a final weight of 25 g. The mixture containing 10% of each nut (100,000 mg/kg) was prepared by adding 2.5 g of the nut flour to 22.5 g of spelt wheat flour, and followed by 10-fold dilutions, homogenizing with the kitchen robot. Moreover, for hazelnut, binary mixtures of DIC treated flours were also created from peanut defatted flour (*Arachis hypogea* L. from Productos Manzanares S.L.), in order to mimic the conditions described in our previous paper, used as a reference for this nut [20].

### 2.4. DNA Extraction and Conventional PCR

The isolation of genomic DNA from nut flours was performed as previously described [21]. Briefly, 100 mg of defatted flour were homogenised in 1 mL of Cetyl trimethylammonium bromide (CTAB) with 1% of polyvinylpyrrolidone (PVP) and 4 µL of 25 mg/mL RNAse, and were incubated at 65 °C for 30 min, shaking each interval of 10 min. The addition of 800 µL of chloroform, mixing, and centrifugation at 13,000 rpm for 10 min were performed. After taking 800 µL of the aqueous supernatant, genomic DNA was obtained following the instructions from the DNeasy Power Plant DNA isolation kit (Qiagen, Hilden, Germany) and eluted in 100 µL of 10 mM Tris pH 8.0. At least two different DNA isolations were carried out. The quantity and quality of the extracted DNA were evaluated on 0.8% TBE agarose gels and using a Nanodrop ND-1000 spectrophotometer (Thermo-Fisher, Waltham, MA, USA), taking into account the values obtained by measuring absorbance at 230, 260 and 280 nm. We also used DNA obtained in our previous studies [20,21,22], from other sequenced varieties of hazelnut (Pauetet, Tonda and Sant Giovanni), pistachio (Aegina, Mateur and Sirora) and cashew (Embrapa 50, BRS 189, BRS 274 and CCP06).

Positive amplification of all samples was tested by PCR using universal eukaryotic primers for 18S described elsewhere (forward 5′-CGCGAGAAGTCCACTAAACC-3′, reverse 5′-CCTACGGAAACCTTGTTACGA-3′) [22]. These reactions were carried out in 20 µL, containing 25 ng of DNA, 250 nM of each primer and 1XFastStar PCR Master Mix (Biotools, Loganholme, Australia). SensoQuest LabCycler (Progen Scientific Ltd., London, UK) was programmed with an initial denaturation step at 95 °C, 5 min, followed by 35 cycles of denaturation at 94 °C for 1 min, annealing at 60 °C for 30 s and elongation at 72 °C for 45 s, and a last step at 72 °C for 5 min.

### 2.5. Targets, Primer Design and Real-Time PCR Conditions

Primers were designed in allergen-coding regions of hazelnut, pistachio and cashew, being conserved among the different varieties (Figure S1), and targets were selected in the Cor a 9, Pis v 1 and Ana o 1 allergen-coding sequences, respectively [20,21,22]. After that, the performance of primers and probes was assayed, regarding specificity, sensitivity and reaction efficiency. Sequences, final assayed, conditions and amplicon size of primers and probes used in the Real-Time PCR assays are summarised in Table 1.

Primers for Cor a 9 allergen-coding sequence were used previously by our group [20], where a real-time PCR assay based on Sybr Green detection method was performed with good results for this target. For the study we present here, a hydrolysis probe has been designed in order to standardize the rest of the assays and as an attempt to improve the sensitivity of the method. In pistachio and cashew, Real-Time PCR-based detection methods were recently published by our group [21,22], selecting Pis v 1 and Ana o 1 as target. In these cases, a Locked Nucleic Acid (LNA, Roche, Basel, Switzerland) probe and a hydrolysis probe were designed and used for pistachio and cashew detection, respectively.

Reactions of amplification were performed in a 7900HT Fast Real-Time PCR (Applied Biosystems, Foster City, CA, USA). In a final volume of 20 µL, 10 µL of TaqMan^®^ Gene Expression Master Mix (Applied Biosystems, Foster City, CA, USA), 0.25 µM (for cashew, pistachio, and hazelnut) or 500 µM (for peanut) of each primer, 0.1 µM (for cashew, pistachio, and hazelnut) or 0.2 µM (for peanut) of each probe, and 5 µL of DNA at different concentrations are added. A final 250 ng of DNA from untreated and treated binary mixtures in spelt wheat and 25 ng of pure DNA from the nuts and wheat were amplified. 

Reactions were run under the following cycling conditions: an initial 10 min step at 95 °C, followed by 40 cycles of denaturation at 95 °C for 15 s and primer annealing and elongation at 60 °C for 1 min. At least two technical replicates and two non-template controls (NTC) were analysed in each plate. Standard curves were obtained with the cycle threshold (Ct) from 10-fold serial dilutions of genomic DNA and from binary mixtures of nut seed flour in spelt wheat. The efficiency of the reaction was calculated from the slope from those standard curves (10^(−1/slope)^ − 1), either Ct vs. log DNA concentration or Ct vs. log quantity of each nut flour.

## 3. Results and Discussion

The influence of DIC treatment on the detectability of target from hazelnut, pistachio, and cashew has been analysed. To determine this, the performance of the reaction was analysed in samples containing known amounts of nut flour in a matrix of *Triticum spelta*. In the case of hazelnut, binary mixtures were also carried out in defatted peanut flour, to mimic the conditions previously performed by Iniesto and collaborators [20].

### 3.1. Effect of DIC Treatment on Hazelnut Detectability

**Figure 1:**
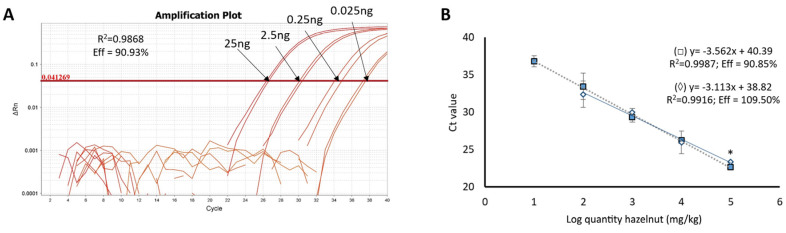
Performance of the amplification reaction for the detection of Cor a 9 target. (**A**) Amplification plot of the calibration curve made from a serial dilution of DNA from Negreta hazelnut variety (*N* = 6). (**B**) Standard calibration curve built with DNA from binary mixtures of a known amount of raw hazelnut in wheat (□) or peanut (◊), representing the log of the mg/kg of the nut in the wheat/peanut matrix vs. Ct value (*N* = 8). * Significant differences among Ct values in the same spiked level between matrix in wheat and peanut (*p* < 0.05), *t*-student unpaired test.

To test the influence of instantaneous controlled pressure drop (DIC) treatment, based on high pressure and temperature for a very short time on the hazelnut allergen detection by Real-Time PCR, the Cor a 9 allergen-coding sequence was the selected target, similar to the study published by Iniesto and collaborators [20]. In contrast to that work, a hydrolysis probe was designed, using the same primer sequences (Table 1), in order to homogenize the three described strategies. The specificity of these primers was already tested [20] and the probe usually confers an increase in the specificity of the method. The standard calibration curve was made with a serial dilution of hazelnut pure DNA from 2500 pg to 2.5 pg, obtaining suitable efficiency in the curve (almost 91%) and correlation coefficient *R*^2^ (Figure 1A). The absolute limit of detection (LOD) was obtained using the analysis of this curve, achieving an absolute LOD of 25 pg. Furthermore, as described before, two binary mixtures with a known amount of hazelnut flour were prepared, one with *T. spelta* wheat flour and other with defatted peanut (*Arachis hypogaea*) flour. Standard calibration curves were built with Ct values against the log of the hazelnut quantity (10^5^ to 10 mg/kg). The parameters of both curves were within the proper criteria (efficiency of 90.85% and 109.50% and *R*^2^ higher than 0.990 for untreated hazelnut in wheat and peanut matrices respectively) (Figure 1B) [23]. In these assays, the curves maintained their linearity up to 100 mg/kg in the mixtures made in peanut and up to 10 mg/kg in those made in wheat (but only in half of the replica). The practical LOD, which is calculated with these standard curves, was 100 mg/kg (since more than 95% of the replica were positive), in contrast to 1 mg/kg obtained by the authors using the intercalant dye Sybr Green as the detection chemistry [20]. However, the Ct values were not delayed in this assay compared to the published one. 

Processing can provoke DNA degradation in different degrees, so these Real-Time PCR methods should be able to reach suitable sensitivity not only in raw samples, but also in treated ones. In order to establish the real influence of any processing, many authors have supported the necessity of analysing the same kind of model mixtures in control and treated samples [24]. For this reason, binary mixtures were also made with DIC treated flours in wheat or peanut flour.

**Figure 2:**
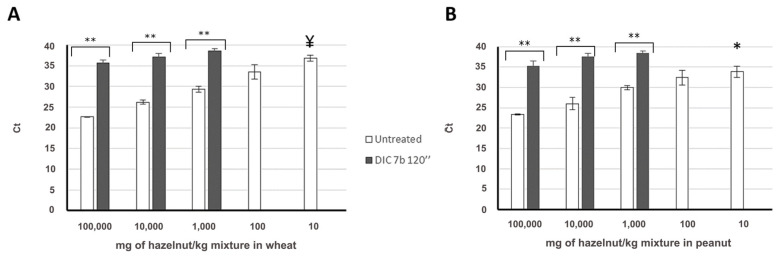
Amplification of Cor a 9 target in untreated (white column, *N* = 8) and treated (black column, *N* = 10) mixtures in wheat (**A**) or peanut (**B**) matrices, representing the quantity of hazelnut in mg/kg of mixture vs. Ct value. ¥ Ct signal only in 50% of the replica. * out of the calibration curve. Mean and standard deviation is shown. Significant differences were found between Ct obtained in untreated and treated samples, for the same spiked level (** *p* < 0.05, by *t*-student test).

The influence of this treatment, DIC 7b 120 s, on Cor a 9 detection in both binary mixtures is shown in Figure 2A,B. In 100,000 mg/kg-containing spiked level, Ct has delayed around 13 cycles compared to untreated sample, and detection of the Cor a 9 target is difficult in mixtures with lower concentrations of hazelnut, being impossible in mixtures with less than 1000 mg/kg (Figure 2 and Figure S2). All Ct values of treated samples were higher than the obtained in samples with 10 mg/kg of untreated hazelnut, and they did not follow a linear regression. This decrease in the Cor a 9 detectability is similar to the exerted by autoclave treatment and described by Iniesto et al. [20].

Iniesto et al. [20] subjected hazelnut nuts to roasting, autoclaving at different conditions, and high-pressure treatments (HHP). DNA was isolated directly from the treated flours, observing that roasting and autoclaving dramatically reduced the extraction yield (in quantity and quality) compared to the control, as well as the capacity of the method for detecting any of the tested targets (Cor a 9, Cor a 11 and Cor a 13). This effect was not observed when hazelnut was subjected to HHP processing, where high pressure but no heat is applied. In the present study, the analysis of the effect of the treatment applied in this study, DIC 7b 120 s, was based on the preparation of binary mixtures in the same way as the control. Consequently, a comparison between our results and the previous data from Iniesto et al. for hazelnut detection is not feasible; however, the effect of DIC treatment on Cor a 9 detectability seems to be very similar to that observed with autoclaving at 138 °C [20]. 

The in vitro immunoreactivity of hazelnut processed by HHP and autoclaving has been previously analysed [25]. Recently, Cuadrado et al. [7] found that HHP treatment applied on hazelnut and almond nuts did not affect its immunoreactivity detected by Western blotting. Regarding treatment, when applying both pressure and heat as in autoclaving methods, the effect was drastic; any allergen was able to be detected after specific conditions of autoclaving. However, as far as we know, the effect of DIC treatment on the IgE/IgG binding capacity of hazelnut allergens has not been studied.

### 3.2. Effect of DIC Treatment on Pistachio Detectability

**Figure 3:**
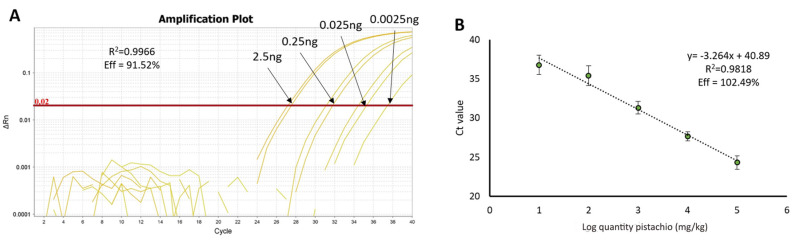
Performance of the amplification reaction for the detection of Pis v 1 target. (**A**) Amplification plot of the calibration curve made from a serial dilution of DNA from Aegina pistachio variety (*N* = 4). (**B**) Standard calibration curve built with DNA from binary mixtures of a known amount of raw pistachio in wheat, representing the log of the ppm (mg/kg) of pistachio in the wheat matrix vs. Ct value (*N* = 8).

In the case of pistachio, the preparation of the binary mixtures in wheat, the DNA isolation method, and the amplification reaction were performed as described by Sanchiz and collaborators [21]. The same primers and LNA probe (Table 1) have been used on this occasion, targeting the sequence Pis v 1, coding for an allergenic 2S albumin. Using the analysis of DNA curves made with serial dilutions of pure nut DNA, from 2500 pg to 2.5 pg, the absolute LOD_s_ was obtained, achieving 2.5 pg. Curves also showed a good performance when taking into account two parameters, *R*^2^ (0.996) and efficiency (91.52%) (Figure 3A). The performance of the curves, when analysing untreated DNA from binary mixtures containing different amount of pistachio flour, was in accordance with the performance obtained previously (*N* = 8), regarding efficiency and *R*^2^, as well as the practical LOD and limit of quantification (LOQ), both 10 mg of pistachio/kg of the mixture (Figure 3B).

**Figure 4:**
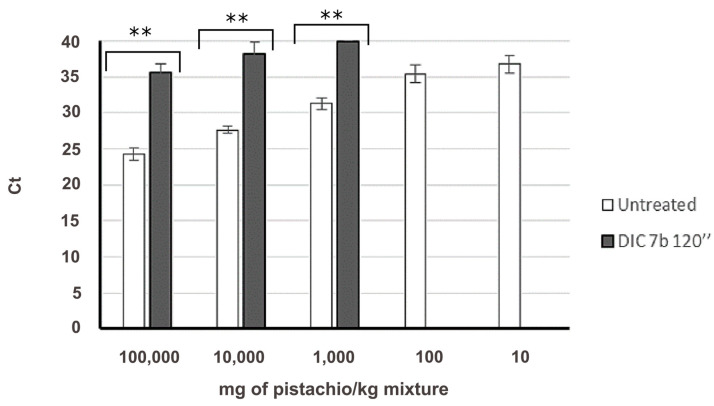
Amplification of Pis v 1 target in untreated (white column, *N* = 8) and treated (black column, *N* = 10) mixtures in the wheat matrix, representing the quantity of pistachio in mg/kg of mixture vs. Ct value. Mean and standard deviation is shown. Significant differences were found between Ct obtained in untreated and treated samples, for the same spiked level (** *p* < 0.05, by *t*-student test).

Regarding the effect of DIC treatment at the particular conditions of 7 bar 120 s on Pis v 1 target, for the same spiked level (100,000 to 1000 mg/kg) of the binary mixtures, Ct is significantly delayed compared to the untreated control, and linear regression was not obtained. In the case of 100,000 mg/kg-containing binary mixtures, the detection was delayed more than 10 cycles (Figure 4). Pis v 1 target was not detectable when treated flour was present in less than 10,000 mg/kg in the wheat mixture (Figure 4). 

**Figure 5:**
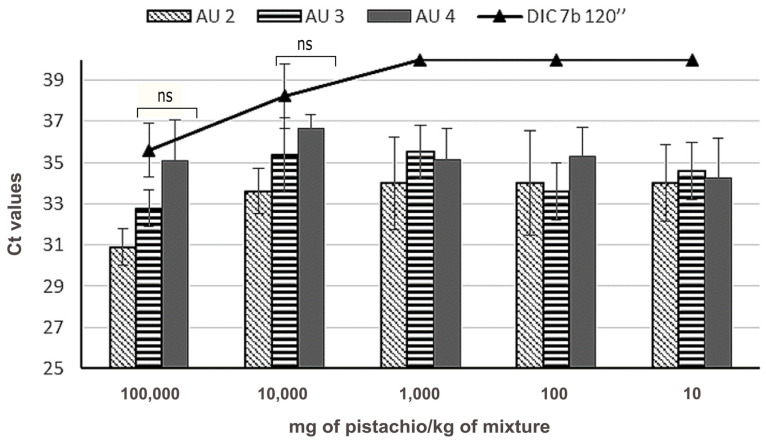
Amplification of Pis v 1 target in binary mixtures of flours treated by instant controlled pressure drop (DIC) 7 b 120 s (line), obtained in this work, and other treated samples by heat and pressure (autoclave (AU): AU 2 (121 °C, 1.18 atm, 30 min); AU 3 (138 °C and 2.56 atm for 15 min); AU 4 (138 °C and 2.56 atm for 30 min) (data from [20]). Mean and standard deviation is shown (*N* = 10 for DIC, *N* = 6 for autoclaves). “ns” means that no significant differences were found between Ct means obtained in DIC and AU 4 samples, at the same spiked level (*p* < 0.05, by *t*-student test).

This result is similar to the effect produced on the detectability of Pis v 1 when pistachio nuts were submitted to heat and pressure in an autoclave, which also combines heat and pressure. These treatments were carried out previously by our group [21] and a direct comparison of data can be performed since sample preparation is equivalent for all treatments, as shown in Figure 5. The effect of DIC 7b 2 min treatment on the detectability of Pis v 1 target was more drastic than the observed in pistachio treated by autoclave treatment at 121 °C for 30 min and 138 °C for 15 min. Differences between Ct mean values in these autoclaved samples and DIC treatment were not significant (data non shown). However, for the more concentrated mixtures (10 and 1% of treated pistachio in wheat), no significant differences between Ct mean values from DIC 7b 2 min and autoclave (AU) at 38 °C 30 min pistachio treated samples were found. 

Recently, Vicente and collaborators [26] studied the effect of DIC 7b 120 s treatment on the in vitro IgE binding capacity of pistachio, using sera from Spanish allergic patients. This treatment allowed the authors to reduce pistachio’s capacity to bind IgG and IgE, although it was able to trigger IgE immunoreactivity in several patients. By mass spectrometry, the authors showed that peptides belonging to two major allergens (Pis v 1 and Pis v 2) survived this treatment. With the Real-Time PCR method we present here, it would be possible to detect traces of this treated pistachio by DIC 7b 120 s (above 1000 mg/kg), guaranteeing the identification of this allergen in processed foodstuff.

### 3.3. Effect of DIC Treatment on Cashew Detectability

**Figure 6:**
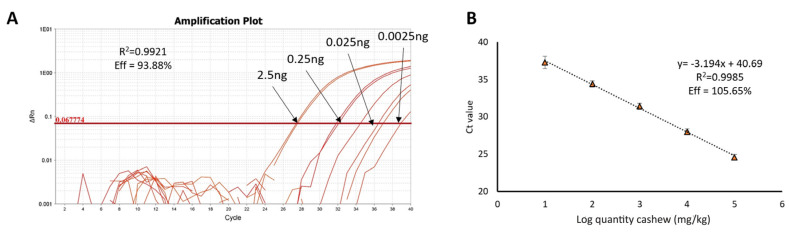
Performance of the amplification reaction for the detection of Ana o 1 target. (**A**) Amplification plot of the calibration curve made from a serial dilution of DNA from raw cashew type 320 (*N* = 4). (**B**) Standard calibration curve built with DNA from binary mixtures of a known amount of cashew in wheat, representing the log of the ppm (mg/kg) of cashew in the wheat matrix vs. Ct value (*N* = 8).

Finally, the influence of this treatment on the amplification of the target Ana o 1 from cashew was studied. Previously [22], we optimised a methodology for the specific and sensitive detection of cashew in complex mixtures, by means of Real-Time PCR and using a hydrolysis probe (Table 1). Two standard calibration curves were built from the Ct values and the log of the cashew quantity. Both curves showed good performance, with parameters of *R*^2^ and efficiency of reaction within the minimal standards for Real-Time PCR publications (efficiency between 90 and 110%, *R*^2^ > 0.99 [27] (Figure 6).

**Figure 7:**
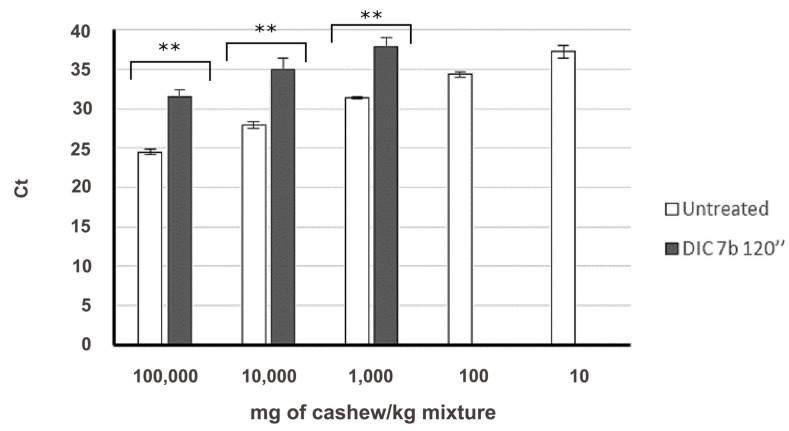
Amplification of Ana o 1 target in untreated (white column, *N* = 8) and treated (black column, *N* = 10) mixtures in a wheat matrix, representing the quantity of cashew in mg/kg of mixture vs. Ct value. Mean and standard deviation is shown. Significant differences were found between Ct obtained in untreated and treated samples, for the same spiked level (** *p* < 0.05, by *t*-student test).

Achieved absolute and practical LODs were 2.5 pg of cashew DNA and 10 mg/kg of cashew flour in the wheat matrix, in concordance with the available data. Specificity was previously assayed by Sanchiz et al. [22]. Figure 7 shows the general effect of DIC treatment, at 7 b for 120 s, on the detection of the Ana o 1 target compared to the control. Again, Ct is significantly delayed compared to the untreated control, up to seven cycles in the 100,000 mg/kg containing the cashew mixture, although with a significantly lower impact than pistachio and especially hazelnut (Figure S3). However, standard curves built with log quantities of treated cashew (mg/kg, just three points) vs. Ct values maintained a good performance with *R*^2^ = 0.9955 and an efficiency of the reaction of 108.84%. Thus, detection and even quantification of the target Ana o 1 was possible even in the samples containing up to 1000 mg/kg of DIC treated cashew flour, in nine out of 10 replicates.

As recently reviewed [11], small amplicon size (<150 bp) is desired since DNA from foodstuff is usually fragmented and it could affect the detectability of the target. In the method for Ana o 1, an improvement compared to other studies that also considered allergen-coding sequences as a target was observed, probably due to the small size of the amplicon (only 65 bp). 

**Figure 8:**
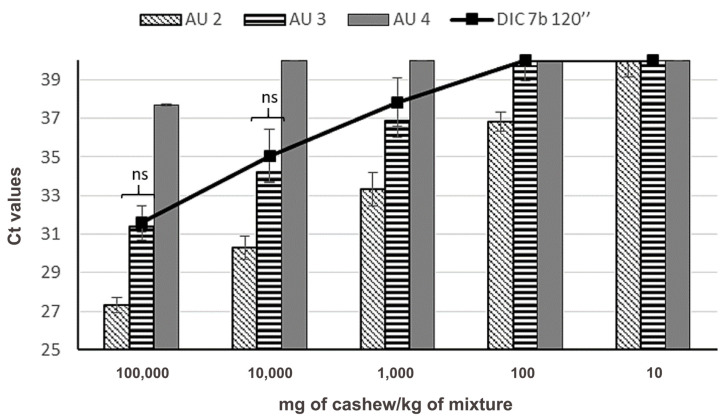
Amplification of Ana o 1 target in binary mixtures of flours treated by DIC 7b 120 s (line), obtained in this work, and other treated samples by heat and pressure (autoclave: AU 2 (121 °C, 1.18 atm, 30 min); AU 3 (138 °C and 2.56 atm for 15 min); AU 4 (138 °C and 2.56 atm for 30 min) (data from [21]). Mean and standard deviation is shown (*N* = 10 for DIC, *N* = 6 for autoclaves). “ns” means that no significant differences were found between Ct obtained in DIC and AU 3 samples, at the same spiked level (*p* < 0.05, by *t*-student test).

Sanchiz et al. [22] reported that this method was very sensitive for cashew detection when it was subjected to heat and pressure at harsh conditions. As described, a statistical analysis of Ct values from autoclaved and DIC treated samples was carried out (*t*-student). At 138 °C for 15 min in an autoclave, the detection and quantification were possible up to 1000 mg/kg, maintaining a linear correlation of the curve (from 10^5^ to 1000 mg/kg) similar to DIC 7 bar for 120 s treatment. Ct means showed no significant differences between both treatments (Figure 8), in contrast to the rest of autoclave treatments when compared to DIC (data not shown). 

As mentioned with pistachio, Vicente and collaborators [26] showed, for the first time, by mass spectrometry, that major cashew allergens (Ana o 2 and Ana o 3) survived to DIC 7b 120 s processing, and triggered IgE immunoreactivity in patients, although a reduction in the capacity to bind IgE was noticeable. This tested Real-Time PCR assay, especially in the case of cashew, allowed the authors to potentially detect traces of the nuts treated by heat and pressure at 7 bar and 2 min, which resulted in the appearance of an immunoreactive response in sensitive patients. In that study [26], pistachio proteins were also more susceptible to DIC processing than cashew ones, and, according to our results, it was possible to detect and quantify treated cashew with good efficiency, much better than pistachio (Figure S3).

Interestingly, the effect of food processing on the capacity to detect traces of a particular allergen has not been deeply studied. As stated before, thermal treatments, with or without the combination of pressure, have a different effect depending on the material, whereas non-thermal treatments, such as HHP, generally have a smaller impact on the detectability of nut targets, as in almond and walnut [28,29]. In general, among thermal treatments, those that combine heat and pressure are responsible for more fragmentation of DNA and make it difficult to amplify the targets in nuts; apart from hazelnut, pistachio and cashew, this has also been demonstrated in walnut, almond, and chestnut [28,29,30]. Treatment based on instantaneous controlled pressure drop (DIC) on foods and its effect on allergenicity has not been extensively analysed. Together with the recent study in cashew and pistachio [26], which have already been discussed, the studies about the influence of this treatment on the IgE reactivity of legumes and milk must be mentioned [31,32]. In the case of legumes [18,19,32], the application of DIC treatment at 6 bar for 3 min did not eliminate the immunoreactive capacity of peanut, chickpea and lentil allergens, but in the case of soybeans and lupin, it was markedly reduced.

## 4. Conclusions

As far as we know, this is the first work studying the effect of DIC treatment, which combines heat and pressure, for a very short time, on the detectability of a DNA target by Real-Time PCR, using hydrolysis probes. We confirmed that the application of pressure, 7 bar and temperature, up to 180 °C, for only 2 min, on tree nut seeds, can reduce the ability to detect allergen-coding sequences from hazelnut (Cor a 9), pistachio (Pis v 1) and cashew (Ana o 1). This effect was less intense in the case of the cashew allergen. According to the available data regarding the effect of DIC processing on the IgG/IgE binding capacity of nuts, this system would allow us to detect traces of DIC 7 b 120 s-processed hazelnut and pistachio, and even quantify treated cashew, if the quantity of the allergenic ingredient was around 100,000–10,000 mg/kg in a food mixture. 

## Figures and Tables

**Table 1:** Primers and probes sequences used for Real-Time PCR assays.

Nut	Name	Sequence (5′→3′)	Final Concentration (nM)	Length (Bases)	Amplicon Size
Hazelnut	Cor a 9 fw	GTCAAAGTGGAAGGCAGGCTT	250	21	101
	Cor a 9 rv	TCCCGCTCTTGCTCACTCTC	250	20	
	Cor a 9 probe	6FAM-ATGGGAGCGACAGGAGAGACAAGAG-BHQ1	100	26	
Pistachio	Pis v 1 fw	GGCAAAGCTCGTACTTCTCCT	250	21	81
	Pis v 1 rv	TCCACAGTAGCGCGGTAGAT	250	20	
	Pis v 1 probe	6FAM-GACGGAAG-BHQ1	100	8	
Cashew	Ana o 1 fw	AGAAAGGACGGGAACGAGAG	250	20	65
	Ana o 1 rv	TTCATCAACGCCACCAGTT	250	19	
	Ana o 1 probe	6FAM-ATGAGGAGGAAGAAGAAGAATGGG-BHQ1	100	24	

## Supplementary Materials

The following are available online at https://www.mdpi.com/2304-8158/9/6/729/s1, Figure S1: Partial alignment of Pis v 1, Ana o 1 and Cor a 9 coding sequences. Figure S2: Amplification of Cor a 9 target in untreated and treated samples included in wheat or peanut binary mixtures. Figure S3: Relative detection of Pis v 1, Ana o 1 and Cor a 9 targets.

## References

[1] Sicherer S.H., Sampson H.A. (2015). Evaluation of Food Allergy. Mount Sinai Expert Guides: Allergy and Clinical Immunology.

[2] Ojeda P., Sastre J., Jm O., Chivato T. (2018). Alergólogica 2015: A National Survey on Allergic Diseases in the Adult Spanish Population. J. Investig. Allergol. Clin. Immunol..

[3] Crespo J.F., James J.M., Fernandez-Rodriguez C., Rodriguez J. (2006). Food allergy: Nuts and tree nuts. Br. J. Nutr..

[4] Flinterman A.E., Akkerdaas J.H., Knulst A.C., van Ree R., Pasmans S.G. (2008). Hazelnut allergy: From pollen-associated mild allergy to severe anaphylactic reactions. Curr. Opin. Allergy Clin. Immunol..

[5] Mendes C., Costa J., Vicente A.A., Oliveira M.B.P.P., Mafra I. (2016). Cashew Nut Allergy: Clinical Relevance and Allergen Characterisation. Clin. Rev. Allergy Immunol..

[6] Costa J., Silva I., Vicente A.A., Oliveira M.B.P.P. (2019). Pistachio nut allergy: An updated overview. Crit. Rev. Food Sci. Nutr..

[7] Cuadrado C., Sanchiz A., Vicente F., Ballesteros I., Linacero R. (2020). Changes Induced by Pressure Processing on Immunoreactive Proteins of Tree Nuts. Molecules.

[8] Cabanillas B., Maleki S.J., Rodríguez J., Burbano C., Muzquiz M., Jiménez M.A., Pedrosa M.M., Cuadrado C., Crespo J.F. (2012). Heat and pressure treatments effects on peanut allergenicity. Food Chem..

[9] Vanga S.K., Raghavan V. (2016). Processing Effects On Tree Nut Allergens: A Review. Crit. Rev. Food Sci. Nutr..

[10] De la Cruz S., Lopez-Calleja I., Martin R., Gonzalez I., Alcocer M., Garcia T., Lin J., Alcocer M. (2017). Recent advances in the detection of allergens in foods. Food Allergens: Methods and Protocols, Methods in Molecular Biology.

[11] Linacero R., Sanchiz A., Ballesteros I., Cuadrado C. (2020). Application of real-time PCR for tree nut allergen detection in processed foods. Crit. Rev. Food Sci. Nutr..

[12] Hird H., Chisholm J., Sanchez A., Hernandez M., Goodier R., Schneede K., Boltz C., Popping B. (2006). Effect of heat and pressure processing on DNA fragmentation and implications for the detection of meat using a real-time polymerase chain reaction. Food Addit. Contam..

[13] Wang J., Vanga S.K., McCusker C., Raghavan V. (2019). A Comprehensive Review on Kiwifruit Allergy: Pathogenesis, Diagnosis, Management, and Potential Modification of Allergens Through Processing. Compr. Rev. Food Sci. Food Saf..

[14] Cabanillas B., Novak N. (2019). Effects of daily food processing on allergenicity. Crit. Rev. Food Sci. Nutr..

[15] Sanchiz A., Cuadrado C., Dieguez M.C., Ballesteros I., Rodriguez J., Crespo J.F., de las Cuevas N., Rueda J., Linacero R., Cabanillas B. (2018). Thermal processing effects on cashew and pistachio allergenicity. Food Chem..

[16] Cuadrado C., Cheng H., Sanchiz Á., Ballesteros I., Easson M., Grimm C.C., del Dieguez M.C., Linacero R., Burbano C., Maleki S.J. (2018). Influence of enzymatic hydrolysis on the allergenic reactivity of processed cashew and pistachio. Food Chem..

[17] Haddad J., Louka N., Gadouleau M., Juhel F., Allaf K. (2001). Application du nouveau procédé de séchage/texturation par Détente Instantanée Contrôlée DIC aux poissons: Impact sur les caractéristiques physico-chimiques du produit fini. Sci. Aliment..

[18] Guillamón E., Burbano C., Cuadrado C., Muzquiz M., Pedrosa M.M., Sánchez M., Cabanillas B., Crespo J.F., Rodriguez J., Haddad J. (2008). Effect of an instantaneous controlled pressure drop on in vitro allergenicity to lupins (Lupinus albus var Multolupa). Int. Arch. Allergy Immunol..

[19] Cuadrado C., Cabanillas B., Pedrosa M.M., Muzquiz M., Haddad J., Allaf K., Rodriguez J., Crespo J.F., Burbano C. (2011). Effect of instant controlled pressure drop on IgE antibody reactivity to peanut, lentil, chickpea and soybean proteins. Int. Arch. Allergy Immunol..

[20] Iniesto E., Jimenez A., Prieto N., Cabanillas B., Burbano C., Pedrosa M.M., Rodriguez J., Muzquiz M., Crespo J.F., Cuadrado C. (2013). Real Time PCR to detect hazelnut allergen coding sequences in processed foods. Food Chem..

[21] Sanchiz A., Ballesteros I., Martin A., Rueda J., Pedrosa M.M., del Dieguez M.C., Rovira M., Cuadrado C., Linacero R. (2017). Detection of pistachio allergen coding sequences in food products: A comparison of two real time PCR approaches. Food Control.

[22] Sanchiz A., Ballesteros I., Marqués E., Dieguez M.C., Rueda J., Cuadrado C., Linacero R. (2018). Evaluation of locked nucleic acid and TaqMan probes for specific detection of cashew nut in processed food by real time PCR. Food Control.

[23] Bustin S.A. (2010). Why the need for qPCR publication guidelines?-The case for MIQE. Methods.

[24] Villa C., Costa J., Gondar C., Oliveira M.B.P.P., Mafra I. (2018). Effect of food matrix and thermal processing on the performance of a normalised quantitative real-time PCR approach for lupine (Lupinus albus) detection as a potential allergenic food. Food Chem..

[25] Lopez E., Cuadrado C., Burbano C., Jimenez M.A., Rodriguez J., Crespo J.F. (2012). Effects of autoclaving and high pressure on allergenicity of hazelnut proteins. J. Clin. Bioinform..

[26] Vicente F., Sanchiz A., Rodríguez-Pérez R., Pedrosa M., Quirce S., Haddad J., Besombes C., Linacero R., Allaf K., Cuadrado C. (2020). Influence of instant controlled pressure drop (DIC) on allergenic potential of tree nuts. Molecules.

[27] Bustin S.A., Beaulieu J.F., Huggett J., Jaggi R., Kibenge F.S.B., Olsvik P.A., Penning L.C., Toegel S. (2010). MIQE précis: Practical implementation of minimum standard guidelines for fluorescence-based quantitative real-time PCR experiments. BMC Mol. Biol..

[28] Prieto N., Iniesto E., Burbano C., Cabanillas B., Pedrosa M.M., Rovira M., Rodríguez J., Muzquiz M., Crespo J.F., Cuadrado C. (2014). Detection of almond allergen coding sequences in processed foods by real time PCR. J. Agric. Food Chem..

[29] Linacero R., Ballesteros I., Sanchiz Á., Prieto N., Elisa I., Martinez Y., Pedrosa M., Muzquiz M., Cabanillas B., Rovira M. (2016). Detection by Real Time PCR of Walnut Allergen Coding Sequences in Processed Foods. Food Chem..

[30] Sanchiz A., Ballesteros I., López-García A., Ramírez A., Rueda J., Cuadrado C., Linacero R. (2020). Chestnut allergen detection in complex food products: Development and validation of a real-time PCR method. LWT-Food Sci. Technol..

[31] Boughellout H., Choiset Y., Rabesona H., Chobert J.M., Haertle T., Mounir S., Allaf K., Zidoune M.N. (2015). Effect of instant controlled pressure drop (DIC) treatment on milk protein’s immunoreactivity. Food Agric. Immunol..

[32] Takács K., Guillamon E., Pedrosa M.M., Cuadrado C., Burbano C., Muzquiz M., Haddad J., Allaf K., Maczó A., Polgár M. (2014). Study of the effect of instant controlled pressure drop (DIC) treatment on IgE-reactive legume-protein patterns by electrophoresis and immunoblot. Food Agric. Immunol..

