# Supplementary material for: Effect of Instant Controlled Pressure Drop (DIC) Treatment on the Detection of Nut Allergens by Real Time PCR
Source: Foods. 2020 Jun 3;9(6):729. doi: 10.3390/foods9060729 (PMC7353585; doi:10.3390/foods9060729)
Supplement: Supplementary file 1 [file foods-09-00729-s001.pdf]

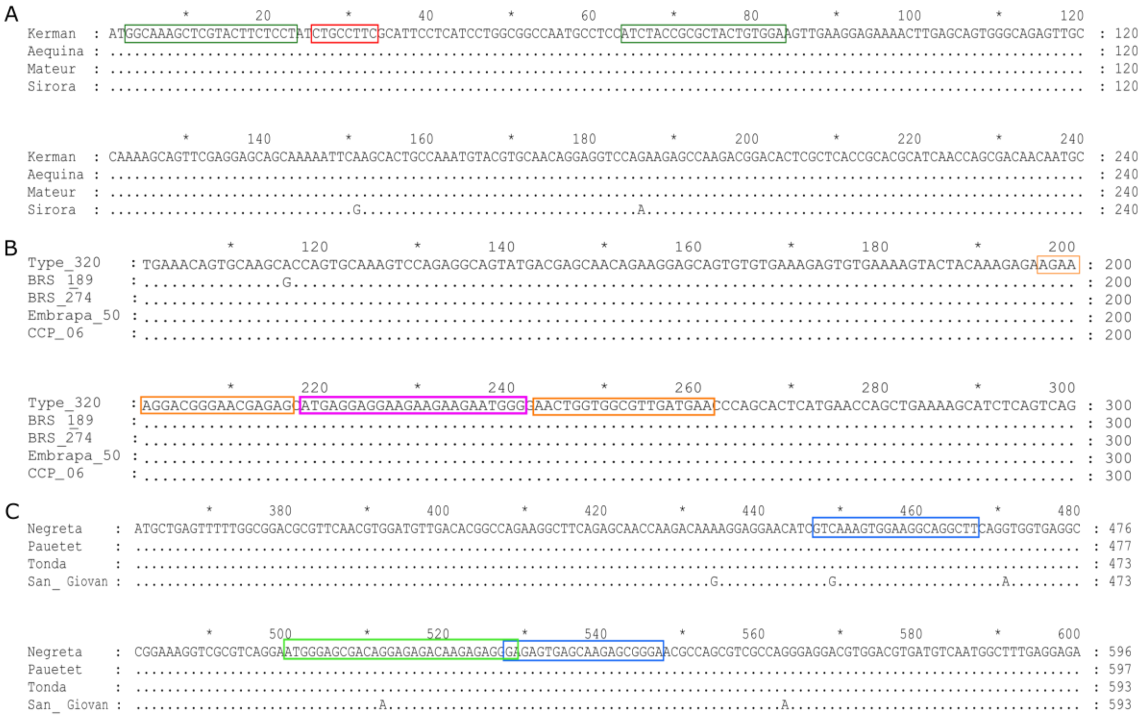

Figure S1. Partial alignment of Pis v 1, Ana o 1 and Cor a 9 coding sequences. Alignment (performed with Clustal Omega) of pistachio (A), cashew (B) and hazelnut (C) varieties of real time PCR targets. Position and sequence of primers and probes are squared. Nucleotide identities are dots in the alignment.

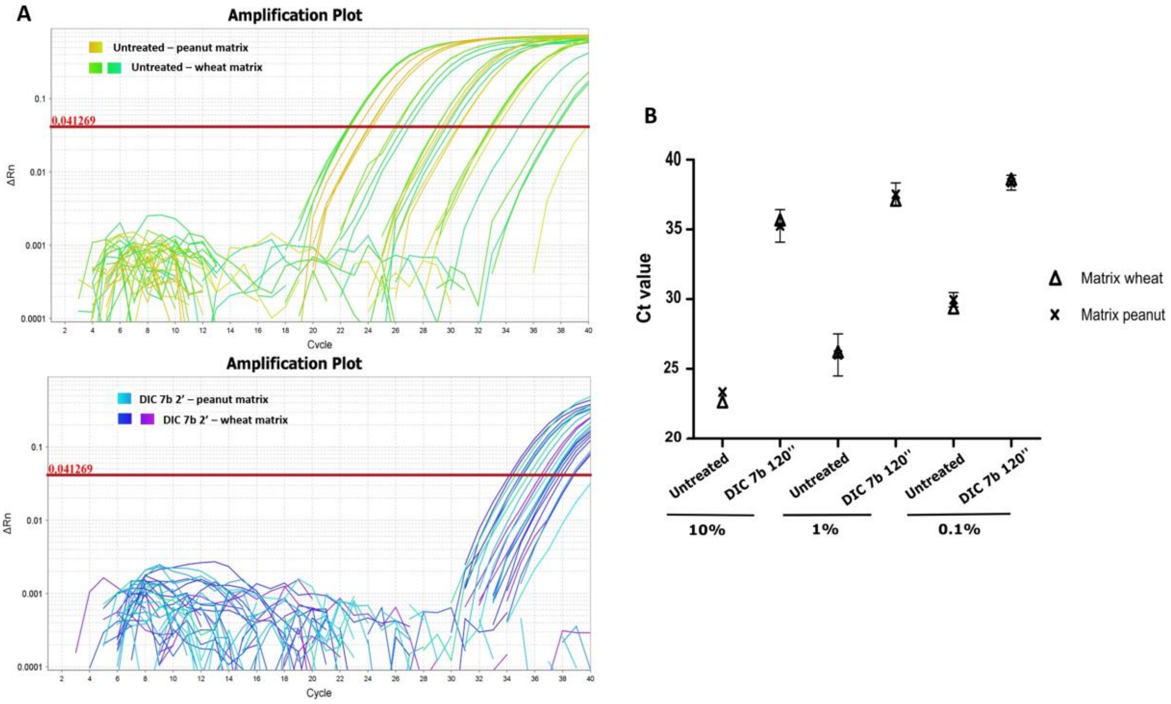

Figure S2. A) Amplification plots of untreated (top) and treated by DIC 7b 120 sec (bottom) hazelnut samples, from 10^5 to 10 ppm of hazelnut in both tested matrices (peanut and wheat). **B)** Ct values for Cor a 9 amplification from binary mixtures in wheat and peanut made with untreated and treated by DIC 7b 120 ''flours. Spiked of higher amount of hazelnut (10% to 0.1%, or 10^5 to 1000 ppm).

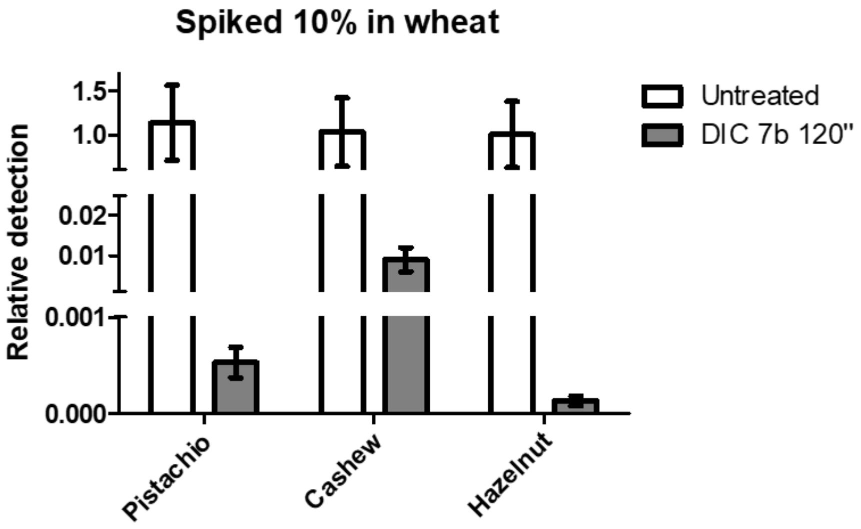

Figure S3. Relative detection of Pis v 1, Ana o 1, Cor a 9 targets in binary mixtures with 10% w/w of untreated and treated by DIC 7b 120'' flours.
